# Spiral Deployment of Optical Fiber Sensors for Distributed Strain Measurement in Seven-Wire Twisted Steel Cables, Post-Tensioned against Precast Concrete Bars

**DOI:** 10.3390/s22197636

**Published:** 2022-10-09

**Authors:** Yanping Zhu, Genda Chen

**Affiliations:** Department of Civil, Architectural, and Environmental Engineering, Missouri University of Science and Technology, Rolla, MO 65401, USA

**Keywords:** distributed fiber optic sensors, post-tensioned concrete structures, cable forces, prestress losses

## Abstract

On-time monitoring and condition assessments of steel cables provide mission-critical data for informed decision making, ensuring the structural safety of post-tensioned concrete structures. This study aimed to develop a spiral deployment scheme of distributed fiber optic sensors (DFOS) and to monitor/assess the post-tensioned force in seven-wire twisted steel cables, based on the pulse-pre-pump Brillouin optical time domain analysis. Each DFOS was placed in a spiral shape between two surface wires of a steel cable and glued to the steel cable by epoxy. Image observations were conducted to investigate the entireness and bonding condition between the optical fiber and the steel wires. Eight concrete bar specimens were cast, each with a pre-embedded plastic or metal duct at its center and each was post-tensioned by a steel strand through the duct once they were instrumented with two strain and two temperature sensors. The strand was loaded/unloaded and monitored by measuring the Brillouin frequency shifts and correlating them with the applied strains and the resulting cable force after temperature compensation. The maximum, minimum, and average cable forces integrated from the measured stain data were compared and validated with those from a load cell. The maximum (or average) cable force was linearly related to the ground truth data with a less than 10% error between them, after any initial slack had been removed from the test setup. The post-tensioned force loss was bounded by approximately 30%, using the test setup designed in this study.

## 1. Introduction

Steel cables are typically used in bridges and prestressed concrete structures, and they have large cross-sectional areas that are subjected to large tensile forces [1]. The rupture of the cable may lead to the progressive collapse of the structures and catastrophic outcomes [2]. For prestressed concrete structures, effective prestressing force, and short-term or long-term prestress losses affect the concrete cracking resistance, deflection, load- bearing capacity, and durability [3]. Therefore, the condition of the cables used in construction and operation directly determines their structural safety and performance. The cable force becomes critical to evaluating the cable and the structural health condition. Measuring the cable force is helpful in monitoring and assessing the health condition of cable-based structures. Different methods have been developed to measure cable forces, including the traditional direct strain measurement method, the oil pressure meter method, the low-cost vibration frequency method, the high-accuracy magnetic flux sensor method in the lab., and acoustic emission technology [4]. Although these methods have achieved great success in cable force measurement in engineering structures, some limitations can be found [5,6,7]. For example, magnetic flux sensors are easily interfered with by electromagnetic fields; indirect vibration-based measurements need to further improve their accuracy and robustness; and strain gauge sensors need temperature compensation, and their long-term stability is a concern, especially in harsh environments. Moreover, many strain sensors are needed to measure forces along the cables (i.e., for a long measurement distance) and the installation of these sensors increases the cost and complexity. In addition, numerous wires for connecting these sensors cannot be handled easily, which affects construction and operation, and even destroys structural performance. Therefore, more effective cable force measurement methods still need to be developed to achieve in situ and on-time monitoring.

In recent years, fiber optic sensors have been used to measure cable forces. The use of fiber optic sensors has many advantages [8,9]. Their very light weight and small dimensions reduce the potential installation effect of fiber optic sensors on the mechanical performance of the cables. Their electromagnetic interference immunity improves measurement robustness and reliability. Moreover, based on Rayleigh and Brillouin scatterings [10,11,12,13], fully distributed strain measurements along the optical fiber have been developed. Fully distributed measurements have the advantages of identifying local interactions that are induced by friction contact between the cable and the duct, and local slip along the cable embedded in the concrete from uneven strain distributions. In the literature, Brillouin-scattering-based distributed sensing technologies have been applied to measure cable forces. Zhou et al. [14,15] developed a smart fiber-reinforced polymer rebar with an embedded optical fiber, and this smart rebar and six wires were bundled as a whole to be used for post-tensioned concrete structures. It has been demonstrated that this smart rebar could monitor prestress loss through the testing of a post-tensioned concrete beam and a prestressed steel frame structure. Although the Brillouin optical time domain analysis (with low spatial resolution) has been applied to achieve a distributed prestress condition assessment, the availability and packaging complexity are limiting smart rebar applications. In addition, the deformation compatibility between the smart rebar and the surrounding wires is required. Once damage occurs at the local area of the smart rebar, the optical fiber may fail to measure prestress loss. The same research group [16,17] used the smart steel strand with a smart rebar, which was instrumented with the optical fiber, to monitor the prestress loss of damaged reinforced concrete structures. The reinforced concrete structures were loaded to the initial cracking stage and the normal service limit state, then they were unloaded to zero. The instant prestress loss during the tensioning process was not identified, and the prestress loss during the unloading process was not emphasized, which means that the prestress reduction due to the removal of the external load was not determined, and the actual prestress loss was, seemingly, unknown. Butler et al. [18,19] used distributed fiber optic sensors (DFOSs) to evaluate the early-age behavior of full-scale prestressed concrete beams. Brillouin optical time domain reflectometry strain sensor cables were installed to the underside of the prestressing strands using plastic cable ties. Although the integrated fiber optic sensing system can be a promising tool for short- or long-term concrete bridge strain monitoring, more details about the installation of these sensor cables to the strands were not given. Webb et al. [20] applied the Brillouin optical time domain reflectometry technique to monitor the strain of a three-span, pretensioned, prestressed, concrete beam-and-slab bridge in the field. The fiber optic cables were embedded in the concrete. The prestressing strands only provided supporting positions because the fiber optic cables were attached to the strands near the ends or to several local points by the tape. Attention was paid to reduce the potential optical fiber cables’ effect on the bond between the sensor cables and the prestressing strands. Moreover, Ansari’s research group [21,22] used the Brillouin optical time domain analysis technique to monitor cables’ tension loss under static and dynamic loading. Both tests were conducted on a scaled cable-stayed bridge, in the lab, and DFOSs were instrumented on the bridge deck. Recently, Li’s research group [23] used the differential pulse-width pair with Brillouin optical time domain analysis to measure strain distributions along the whole length of a 1108 m suspension bridge, and the bridge design was evaluated by the measurement data. Overall, the use of DFOSs, based on the pulse-pre-pump Brillouin optical time domain analysis, to directly measure cable forces is still lacking and is not comprehensive.

As an emerging new cable force measurement technology, the distributed sensor design and installation needs to be clarified and validated. To advance the innovative application of DFOS, based on Brillouin scattering, this study proposes a spiral optical fiber deployment scheme to measure cable forces in concrete during tensioning processes (i.e., before and after prestressing force release). The experimental test setup, the DFOS instrumentation scheme, as well as the tensioning system and the procedures are described in the next section of this paper. The proposed cable force measurement effectiveness was demonstrated by comparing the collected DFOS data with the load cell values. The relationships between the optical fiber-measured results and the ground truth cable force were established, and the instant prestress loss for the present test setup was identified from the distributed fiber optic sensing data. This study provides a new deployment scheme to measure cable forces by using distributed fiber optic sensing technology.

## 2. Experimental Program

### 2.1. Material Properties

**Figure 1:**
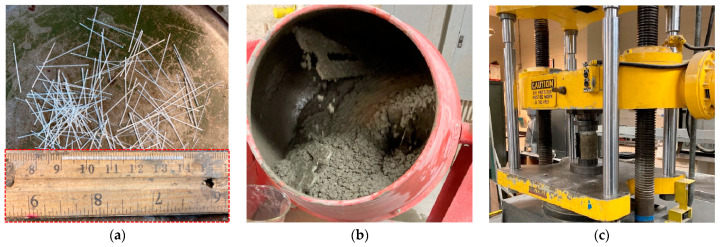
(**a**) Fiber appearance and fiber length of 50 mm; (**b**) concrete mixture in a mixer with a capacity of 170 L; and (**c**) compressive test for cylinder concrete specimen.

The concrete used in this study was designed in accordance with the mix proportions shown in Table 1. The concrete included cement, fly ash, river sand, coarse aggregate, air-entrained agent (DAREX^®^ AEA, GCP, USA), high range water reducer (HRWR-ADVA 198), and viscosity-modifying admixture (CONCERA CP 1124). Among them, the coarse aggregate—with a size range between 9.5 and 12.7 mm—was from the Capital Quarry, Sullivan, Missouri, and the riverbed siliceous sand was from the Capital Quarry, Jeff City, Missouri. The macro fiber (brand name: STRUX BT50) was mixed in the concrete. It had a fiber length of 50 mm and aspect ratio of 75, as shown in Figure 1a. Its tensile strength and elastic modulus were 550 MPa and 7 GPa from the supplier. Other information related to the macro fiber refers to Table 2. The specific mix procedures for concrete were as follows: the coarse aggregate and fiber were added to the sand for mixing, 2 min. Next, half of the water, mixed with AEA, was added to the mixer, mixed for over 1 min. After this, the cement and fly ash were added, and the mixing continued for another 1 min. Afterwards, the remaining ¼ of the water, mixed with ¾ of the HRWR, was added to the mixer and mixed for over 1 min, and the remaining ¼ of the water, mixed with the VMA, was added for another 3-min mixing. The final ¼ of the HRWR was used to adjust the mixture fluidity (i.e., slump flow over 500 mm). Before the concrete was ready for casting, the concrete was mixed for another 2 min. Note that the fiber was gradually added during the whole mixing procedure to guarantee a relatively uniform distribution.

Figure 1b shows the concrete mixture ready for use. The concrete compressive test was performed as shown in Figure 1c. The cylinder specimen dimensions were 100 mm diameter by 200 mm length. The tested average compressive strength was 52.5 MPa. For prestressing strand used in this study, the nominal diameter of the seven-wire steel strand was 12.7 mm, and the nominal cross area was 98.7 mm^2^. The mass/meter ratio was 0.88 kg/m. The ultimate tensile strength was 1860 MPa and the elastic modulus was 200 GPa. Furthermore, the center wire diameter was 4.3 mm and the outer wire diameter was 4.2 mm.

### 2.2. Specimen Design and Preparation

**Figure 2:**
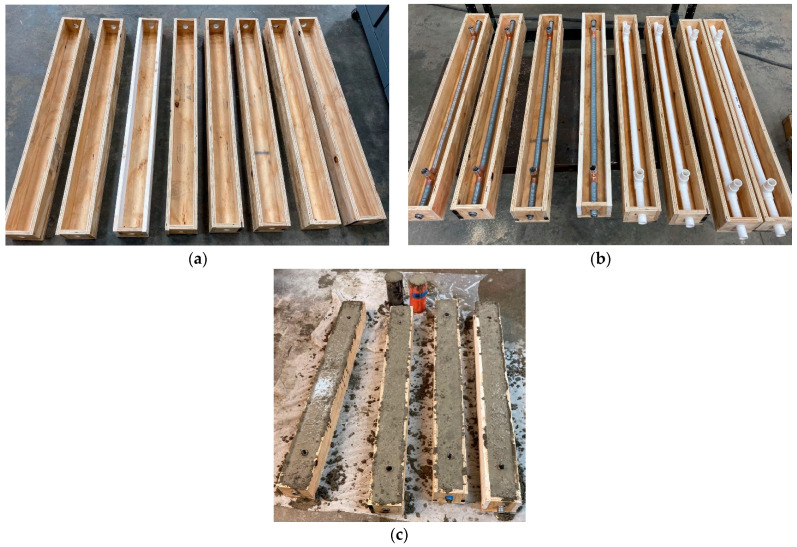
Specimen fabrication: (**a**) mold assembly; (**b**) plastic pipe and corrugated steel duct for post-tensioning strand and grouting; and (**c**) casting specimens.

Eight post-tensioned, prestressed concrete specimens were designed, and all concrete specimens had the same dimensions. The specimen dimensions were 1.219 m long, and 100 mm by 100 mm cross-section. A 25.4-mm-diameter duct was reserved at the center of the cross-section of the specimens, as shown in Figure 2. Moreover, two tee sockets (i.e., pipe fittings) were used in each specimen for connecting the pipe segments as a whole and the vertical opening could be used for grouting later. The test parameters (see Table 3) included bonded/unbonded prestressing strands, presence of paper tape defects and duct materials (plastic and metallic), as well as different adhesives for bonding DFOSs to the strand. In this study, the aim was to measure the cable force using DFOSs during the tensioning process. Therefore, the effect of these parameters on the prestressing force change could be neglected. However, the effect of these parameters on the force change after grouting and under high temperatures is reported later. In the process of concrete casting, a rebar was inserted into the duct to provide sufficient supporting force and keep the duct straight. Figure 2c shows concrete casting into the molds. During demolding, the supporting rebar was taken out. Afterwards, they were moved to a standard curing room before post-tensioning the steel strands. The steel strands with desirable length were cut for DFOS instrumentation and the surfaces of the concrete specimens were grinded and cleaned before post-tensioning the steel strands.

### 2.3. Distributed Fiber Optic Sensing Principle

**Figure 3:**
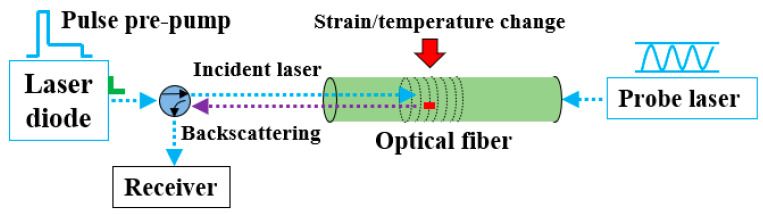
PPP-BOTDA working principle.

Pulse-pre-pump Brillouin optical time domain analysis (PPP-BOTDA)—stimulating the phonon with a long-duration pulse, before a short-duration pulse arrives—measures temperature and strain changes by relating them to the change in the refractive index of an optical fiber and the speed of acoustic waves traveling along the optical fiber. As shown in Figure 3, a probe continuous wave and a pump pulse wave, counter-propagating, were sent from two ends of an optical fiber. Once the frequency difference between the continuous and pulse waves matched the optical fiber Brillouin frequency, Brillouin loss or gain occurred, which was associated with the fiber medium density. The density and refractive index are affected by both strain (ε) and temperature (T), while the Young’s modulus and Poisson’s ratio are affected by temperature (*T*) only [24,25]. Therefore, strain and temperature changes in the optical fiber cause a shift in the Brillouin frequency. For a change in strain and temperature from the reference values obtained during calibration, the Brillouin frequency shift $(\Delta v_{B}$) can be expressed as follows:

(1) $$\Delta v_{B}=C_{\varepsilon }\Delta \varepsilon +C_{T}\Delta T$$

where $C_{\varepsilon }$ and $C_{T}$ represent the strain and temperature sensitivity coefficients, respectively. Spatially distributed Brillouin gain spectra were measured along the length of the tested single mode optical fiber using a Neubrescope data acquisition system (Model NBX 7020), from which the Brillouin frequency was determined, using a Lorentz curve-fitting algorithm. Because the tensioning process was conducted at the constant lab environment, the temperature change was considered zero. Therefore, only the first term (i.e., strain change) induced the Brillouin frequency shift. In this study, an optical fiber with a buffer layer (in addition to the outer coating, inner coating, cladding layer, and glass core) was used for sensing strain. From the lab calibration test (i.e., uniaxial tensile test), the strain frequency shift coefficient for the optical fiber was 19,570.1 με/GHz, at ambient temperature. PPP-BOTDA has a high spatial resolution of 2 cm (at 0.2-ns pulse width) and kilo-meter order measurement distance, which is better than the spatial resolution of 15 cm, or even larger, in the traditional BOTDA and Brillouin optical time domain reflectometry (BOTDR). Moreover, 15 με and 0.75 ℃ measurement accuracies can be, respectively, achieved for strain and temperature with an average count of 2^15^ in PPP-BOTDA.

The Neubrescope NBX-7020 (Neubrex Co., Ltd., Kobe, Japan) data acquisition system can achieve both Brillouin and Rayleigh scatterings. The measurement accuracies of Brillouin scattering can potentially be improved by approximately 7 times in temperature and 15 times in strain, using Rayleigh scattering. However, the cross-correlating, two-by-two measurement approach is needed to increase measurement accuracy by correlating the current measurement with the immediately past reference (i.e., zero point) for Rayleigh scattering. Due to the requirement for cross correlation between any two sequential measurements, the applied strain steps (several tens of microstrains) or temperature steps must be sufficiently small to ensure accurate measurement of a corresponding minimal frequency difference in Rayleigh scattering. The overall strain or temperature effect can be accumulated by summing all steps. When the strain difference exceeds 500 με, the cross correlation in the tunable wavelength coherent optical time domain reflectometry (TW-COTDR) is prone to fail, as verified experimentally in previous studies. Therefore, Brillouin sensor was selected for prestressing force monitoring in this study, since prestressing force usually induces large strain in the steel wires during tensioning process.

### 2.4. Instrumentation and Loading

**Figure 4:**
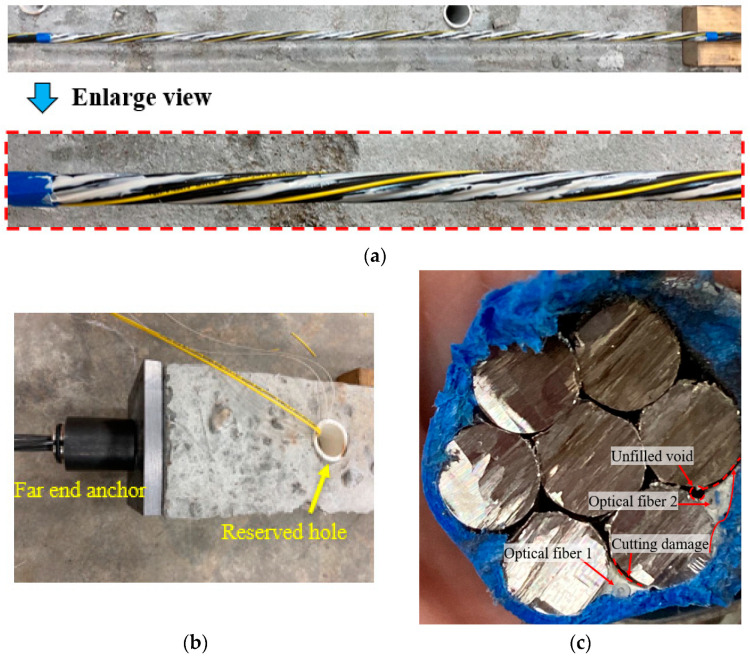
(**a**) Distributed fiber optic sensors’ instrumentation on the steel strand; (**b**) DFOS taken out from the reserved holes; and (**c**) cross-section of a steel strand with optical fibers.

Two types of DFOS were instrumented on each steel strand. One was supposed to measure the strain and temperature simultaneously, while the other was supposed to measure the temperature only. Two optical sensors were prepared for each type to avoid unexpected damage, and to increase surviving rate and data repeatability. These DFOSs were kept helix along the valley between the adjacent outer surface wires and parallel along the outer wires. Using the spiral DFOS shape was beneficial in avoiding easy debonding and fracturing, compared to a straight instrumentation scheme. A small tension was applied during installation, which would have caused initial strain condition. However, this initial condition may not have affected the measured results because it was used as the reference for later measurement results. Figure 4a shows these DFOS arrangements along the strand. The paper tapes were used to fix the temperature sensors along the strand. However, liquid glue was first applied to the strain sensors. Next, a two-part epoxy (i.e., Loctite brand, USA) was used to bond the strain sensors to the strand and at least 24 h were needed for the epoxy hardening. Note that a hi-purity alumina adhesive (supposed to have high temperature resistance) from MTI Corporation, USA, was used for PC5 specimen to see the effect of the adhesive type used on the measured results. In Figure 4, the bond length to the steel strand can be seen, which was not equal to the center distance of the two small holes (see Figure 2). A small reduction from the center distance (approximately 1 cm from each end) can be observed, facilitating taking the DFOS out from the duct later and reducing DFOS damage risk during tensioning. Before the strand that was instrumented with DFOS was put inside the duct, the small concrete pieces inside the duct, which came from demolding and surface cleaning, were cleaned. The steel plates, with a thickness of 12.7 mm, were bonded to the specimen ends using a two-part epoxy (i.e., tank bond brand or Loctite brand, USA) to reduce the stress concentration at the anchor location after prestressing force release. At the same time, the two ends of each fiber sensor were numbered for later splicing and easy distinguishment, because two ends are needed for PPP-BOTDA measurement (i.e., probe and pump ends). Careful attention should be paid when placing the strand, instrumented with DFOS, inside the duct to avoid any damage and fracture, especially for the strain sensors with less protective layers. However, the friction between the strand and the duct surface cannot be avoided due to the weight and length of the strand during the placement. After the strand was carefully placed inside the duct, the DFOS lead portion was taken out from the small, reserved holes using a fishing hook, as shown in Figure 4b, to avoid damage caused by anchoring the strand. It would be very tight and DFOS damage may occur if the DFOS went along the strand.

To observe the bond between the optical fiber and strand in a cross-section, a segment of steel strand that was instrumented with two DFOSs and covered by the epoxy was cut from a long specimen sample, which was loaded to 57.8 kN first (approximately equal to second load level) and unloaded. Figure 4c shows the cross-section cut from the sample. Several layers of paper tapes and two clips were used to fasten the strand at the cutting area and to avoid wire rotation, since the cutting process may induce stress release in these twisted wires. Although this effort was made, the mechanical damage can still be observed, as highlighted by the red dash lines in Figure 4c. Some findings from these image observations are as follows: The epoxy had a good bond to the steel strand, in addition to the mechanical damage. Since the optical fiber was placed at the valley between the adjacent two wires first and the viscous epoxy covered the optical fiber, optical fiber 1 was encased by the epoxy due to the fluidity, while an unfilled void can be observed at the bottom triangle region, where the epoxy could not arrive near optical fiber 2. Any movement of the optical fibers during the covering process affected final optical fiber position and epoxy fullness on the cross-section. Therefore, the position uncertainty and the void defect (probably distributed along the strand) from the optical fiber installation were responsible for the measured errors of the prestressed forces from DFOSs, as compared with the load cell values.

**Figure 5:**
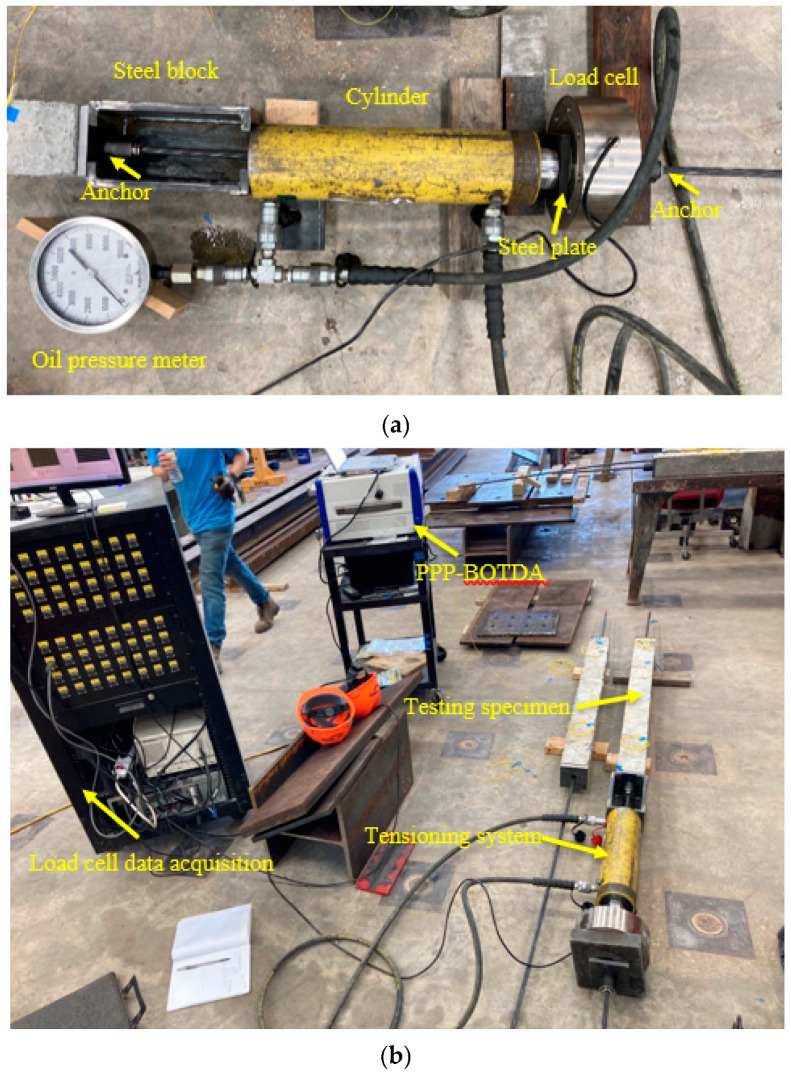
(**a**) New tensioning frame and (**b**) overall test setup.

The test setup for applying prestressing force is shown in Figure 5. A costume-designed steel block for tensioning was used. A hydraulic jack, with a maximum capacity of 30 tons and an effective contact area of 4658 mm^2^ (7.22 in^2^) for the cylinder, was used. The specific tensioning procedure was as follows: First, the far end of the steel strand was anchored. After the load cell was installed at the tensioning end, as shown in Figure 5a, the prestressing force was manually applied by the cylinder. Once the target prestressing force value was reached, a hammer was used to solidate the anchor chucks at the tensioning end, trying to make a tight anchoring and reduce prestress loss once the force was released. However, the instant prestress loss due to the anchor retraction, steel plate and epoxy deformation, as well as elastic deformation of the concrete specimen could not be avoided. After the force was released (less than 10 s) by the cylinder, the tensioning process was finished. Note, that during the tensioning process the concrete specimen was not subject to any applied force and the prestressing force was applied on the specimen after anchoring and force release. Three (or four) load steps were performed, namely 6.5%, 32.5%, and 65% (and 75%) of the ultimate strength of the steel strand. At the final load step, an extra 5% force was applied to improve the prestress level in the strand after instant prestress loss. At each load level, several-time DFOS measurements were conducted, and the oil pressure meter readings were also recorded. For the load cell, its readings could be automatically recorded from the beginning. However, after the force release, the oil pressure meter and load cell did not work, and the prestress force on the cable could be monitored by the DFOSs in this test setup, which highlights the advantage of the DFOS technology to measure on-time cable force distribution and long-term prestress loss due to concrete shrinkage and creep. Figure 5b shows the overall test setup for one testing specimen. Note, that the oil pressure meter was only used for specimen PC1. A brief discussion for the DFOS survivability at the high load may be of interest to the readers. From Gao et al.’s study [22], the optical fiber bonded to the steel strand could measure the stress of approximately 1100 MPa (out of 1860 MPa), although the nominal cross-sectional area of the strand was approximately 140 mm^2^. In their conclusion, they suggested using 60% of 1860 MPa as the tensioning force. However, in this study, the tensioning force was extended to 65% or 75% of the ultimate strength of the strand, and it was found that the DFOSs covered by the epoxy survived and functioned after tensioning at ambient temperature.

## 3. Results and Discussion

### 3.1. Cable Force during Tensioning Process

**Figure 6:**
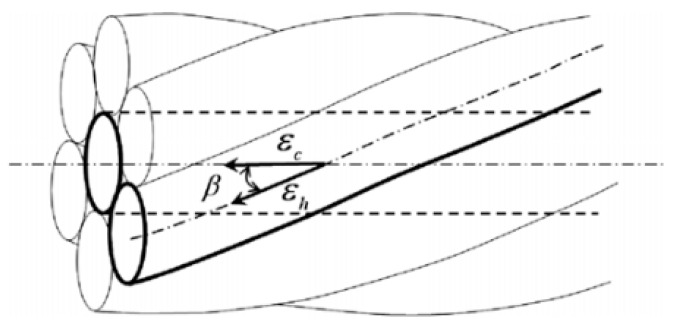
Strain relationship and lay angle [28]. Reprinted/adapted with permission from Ref. [28]. 2010, Elsevier.

As mentioned in Section 2.4, distributed fiber optic sensing measurements were performed at each load step during the tensioning process. The load level applied was also recorded by the load cell. Two distributed fiber optic strain sensors were helically bonded to the strand in each specimen. The measured results in the PPP-BOTDA data acquisition system were Brillouin frequencies. Therefore, the initial Brillouin frequency was needed as a reference for calculating the actual Brillouin frequency shift caused by the prestressing force. The initial Brillouin frequency was recorded, since the value was not zero due to the epoxy shrinkage during the hardening and the restraint effect provided by the strand, which can be seen in the later discussion. After the Brillouin frequency was subtracted by the initial condition value, Equation (1) was used to convert the frequency shift to the strain of the optical fiber. The second term in Equation (1) was neglected due to the constant temperature environment in the Highbay Laboratory, Missouri S&T, Rolla, MO, USA. Here, it was assumed that the strain transfer between the optical fiber and the steel strand was perfect because the two-part epoxy was used for bonding the optical fiber to the strand [26]. Therefore, the strain obtained from the optical fiber can be regarded as the strand strain. As the optical fiber was bonded to the strand at the valley along the outer wires, the strain calculated from Equation (1) was an average strain of the adjacent two wire strains. Based on this discussion, the method for calculating prestressing force is provided below, which has been used in previous studies [27,28]. Equations (2) and (3) show the formulas for the prestressing force calculation with a lay angle, as follows:

(2) $$\varepsilon_{c}=\frac{1}{cos^{2}\beta }\varepsilon_{h}$$

(3) $$f_{p}=A_{p}\times \varepsilon_{c}\times E_{p}$$

where, $\varepsilon_{c}$ is the strain of the center, $\varepsilon_{h}$ is the strain of the helical wires, and β is the lay angle, as shown in Figure 6. The strain of the helical wires ($\varepsilon_{h}$) is the measured strain from the optical fiber. Different strain statistics can be chosen to calculate prestressing force for comparison purposes because the strain distribution along the strand was obtained, which highlights the technical advantage of distributed fiber optic sensing in data collection. For example, average strain, maximum strain, and minimum strain from the strain distribution along the strand can be used as the strain of the helical wires ($\varepsilon_{h}$). By comparing the calculated prestressing forces with the load cell values, the errors between them can be identified, and the distributed sensing effectiveness to monitor prestressing force can be examined, which was also the aim of the present study. In the following subsections, the measured and calculated results (strain and force) are presented for each specimen. Because many data were obtained from the distributed sensing, it is not ideal to combine them together to reduce analysis complexity.

**Figure 7:**
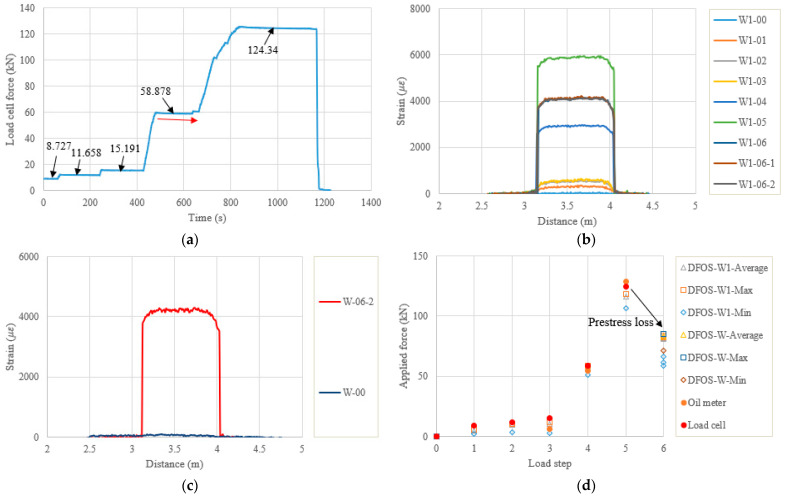
(**a**) Calibrated load cell readings for PC1; (**b**) strain distribution along the strand from one strain fiber (W1); (**c**) the other fiber (W); (**d**) applied force changes at different load steps.

Figure 7 shows the applied force history measured by the load cell, the strain distributions along the strand, as well as the calculated prestressing forces for PC1. There are six load steps, as can be observed in Figure 7a, and the initial load step was not recorded for the first specimen. As the force was applied by the jack cylinder, the force monitored by the load cell could not maintain a stable and constant value after the target force value was reached at each step. The target force gradually decreased, as can be seen in Figure 7a. Therefore, the values written on Figure 7a represent the average forces during each load step, when the distributed sensing measurements were performed. Figure 7b,c show the measured strain distributions along the outer wires of the strand, representing two instrumented optical fibers (namely, W1 and W). However, the W optical fiber only had the initial value and strain distribution after the force release. Generally, the strain distributions along the wires at each load step are uniform, and a strain gradient can be seen at the two ends, which may be attributed to the strain transfer at the ends. In addition, the repeatability for the two-time measurements at each step is acceptable. The maximum strain measured by the DFOSs was approximately 6000 με and the strain after the force release was approximately 4000 με. Figure 7d shows a comparison of the applied force obtained for the different measurement systems. The force from the oil pressure meter was calculated by the meter reading, multiplied by the effective contact area of the cylinder, while the DFOSs’ forces were obtained by the average strain, maximum strain, or minimum strain (i.e., distance from 3.255 m to 3.942 m for the W1), multiplied by the material properties and the geometrical angle of the strand. The oil pressure meter did not have readable values at the first two steps, while the load cell had an initial value of 0.205 kN. At the third step, the pressure meter only measured the force of 6.423 kN, which was much smaller than the load cell and DFOS values of 15.191 kN and 10.347 kN (from W1 average strain, the same source used for the following comparisons), probably because of the gaps in the test setup and manual reading of the pressure meter. At the fourth step the three values approached 58.878 kN, 54.597 kN, and 57.741 kN, and at the fifth step they approached 124.340 kN, 128.464 kN, and 116.364 kN, for load cell, pressure meter, and DFOS, respectively. Overall, the three values had a good consistency. For all the DFOS values, they were smaller than the load cell values because the strain transfer between the optical fiber and strand may exist. Another reason is that the center wire of the strand has a higher strain than the outer wires in the strand axis direction [29]. In the present calculation, the latter is used for calculating cable force. For the prestress loss calculated from the DFOSs after the force release in PC1, the actual average prestressing force value (from W1 average strain) was 81.000 kN, which was reduced by 30.4% compared to the last step. This instant prestress loss was probably due to the gap between the specimen end and the steel plate (approximately 1 mm for PC1), the epoxy deformation, and the concrete elastic deformation. Therefore, two improvement methods for reducing prestress loss are proposed. The first is to improve the final tensioning force, however, attention should be paid to the end compression zone during force release. Although the force after the prestress loss that is applied on the concrete specimen is usually acceptable, the sudden impact effect of the large tension force during the force release (less than 10 s) on the concrete specimen needs attention. The second is to improve the anchoring quality. In this study, increasing the hitting times for the tensioning anchor before the force release was beneficial. Furthermore, trying to reduce the gaps between the anchor, the specimen, and the end steel plate, and aligning more perfectly between the cylinder and the concrete specimen would be helpful.

Table 4 lists the quantitative comparisons between the measured and calculated forces. Note that, the small initial values of the load cells and the DFOSs were subtracted by their readings at a specific load to obtain the applied force-induced changes. The initial value of the load cell may come from the drift error, while the initial value of the DFOS comes from hardening epoxy deformation, as described before. The relative error was calculated by (DFOS − load cell)/(load cell) × 100%, which also applies to the other tables. It was found that, at the lower loads (the first three steps for PC1), the errors between them were up to 42.69%, 33.12%, and 81.15% for the DFOS average, DFOS max., and DFOS min., respectively. As the applied force increased, the errors significantly decreased. The errors between the load cell and the DFOS max. were less than 5%, while the errors for the DFOS min. were approximately 14%. Moreover, the prestressing force could only be monitored by the DFOSs after the force release in this test setup because of the load cell availability.

**Figure 8:**
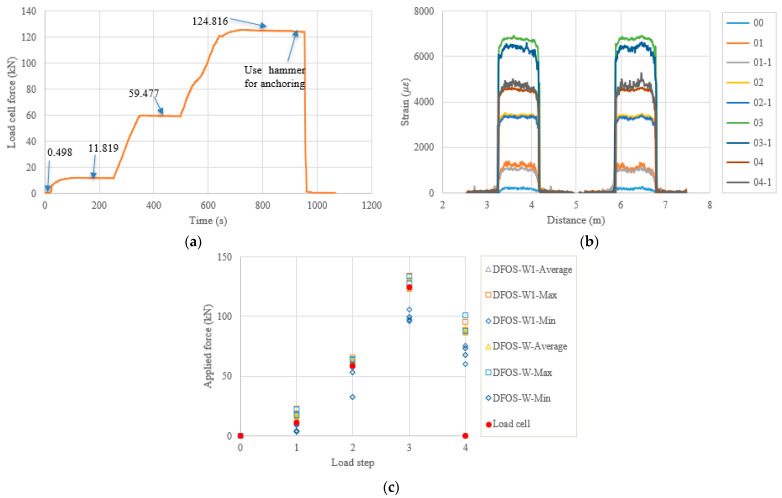
(**a**) Load cell readings for PC5; (**b**) strain distribution along the strand (W1 and W fibers) (In the legend, the first number represents the load step, and the second number represents measurement times at the load step.); (**c**) applied force changes at different load steps.

**Figure 9:**
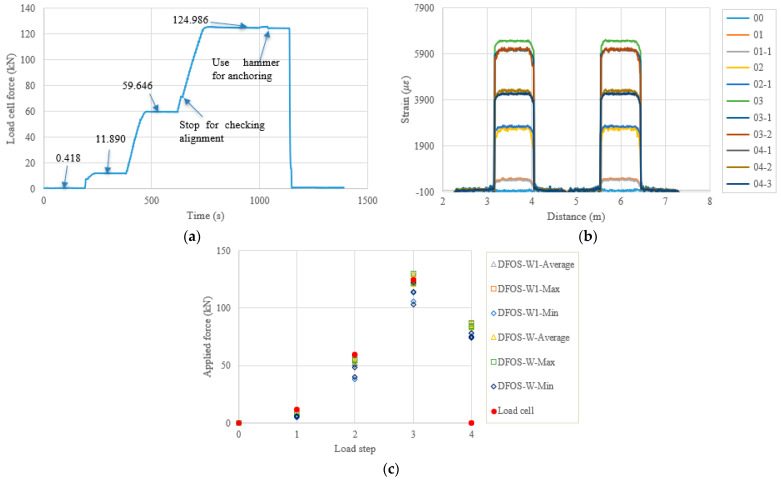
(**a**) Load cell readings for PC6; (**b**) strain distribution along the strand (W1 and W fibers) (In the legend, the first number represents the load step, and the second number represents measurement times at the load step); and (**c**) applied force changes at different load steps.

**Figure 10:**
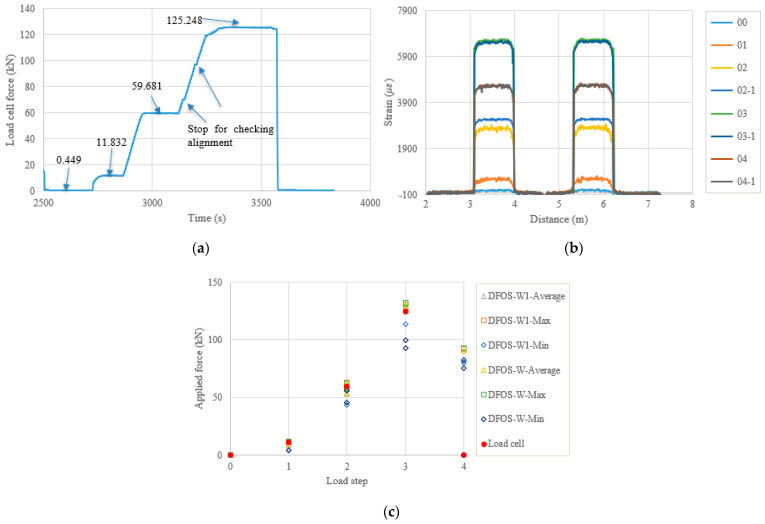
(**a**) Load cell readings for PC2; (**b**) strain distribution along the strand (W1 and W fibers) (In the legend, the first number represents the load step, and the second number represents measurement times at the load step); and (**c**) applied force changes at different load steps.

In the following content, the figures and tables, such as Figure 7 and Table 4, are presented for the remaining specimens in accordance with the test date. Because the specimens followed almost the same tensioning and measuring procedures, the general discussion and data presentation are the same as those in PC1. Here, the authors try to highlight different aspects in different specimens. Figure 8 and Table 5 show the test and calculated results of the PC5 specimen. The high temperature adhesive was used for PC5, which was different from that used for the other specimens, for which the normal ambient temperature epoxy was used. Furthermore, the hammer was used for anchoring before the force release and there were four load steps. The two optical fibers were connected in one loop to reduce the measurement time and the distributed strains could be plotted in one figure. From Figure 8b, it can be seen that there are measurement peaks (i.e., less smooth in PC5), which may have come from the adhesive surface cracks and are different from the other measurements. This observation indicates that the adhesive affected the final measured results, and that additional analysis is warranted. In Table 5, the errors between the load cell and the DFOS values for the average and max. cases in the two optical fibers were less than 10% after the second load step, while for the minimum case, the errors were up to approximately 20%. Figure 9 and Table 6 show the testing results of the PC6 specimen. For this specimen, before the hammer was used for anchoring, the applied load was increased to the target value to slightly compensate the load reduction with time, since the force could not be applied after the tension end chuck was tightly anchored. Furthermore, a slight force fluctuation can be seen because the hammer hit for anchoring and this operation did not affect the applied force prior to the force release. Moreover, a stop point is observed in Figure 9a, to check the alignment of the loading system during the tensioning process. Again, from Table 6 it can be seen that the errors between the DFOS-measured cable forces and the load cell forces were within 10% for all average and max. cases. Figure 10 and Table 7 show the testing results of the PC2 specimen. It was observed that the first measured DFOS values were smaller than the second ones, at the second step from Figure 10b, while the opposite trend is true for the PC6 specimen at the third step (see Figure 9b). From Table 7 it can be seen that the maximum errors for the DFOS-min. case were up to approximately 25%, except for the first load level.

**Figure 11:**
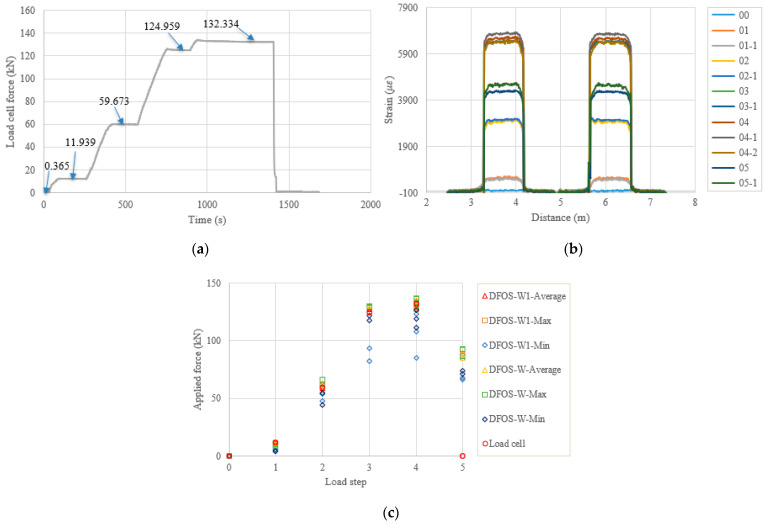
(**a**) Load cell readings for PC3-1; (**b**) strain distribution along the strand (W1 and W fibers) (In the legend, the first number represents the load step, and the second number represents measurement times at the load step); and (**c**) applied force changes at different load steps.

**Figure 12:**
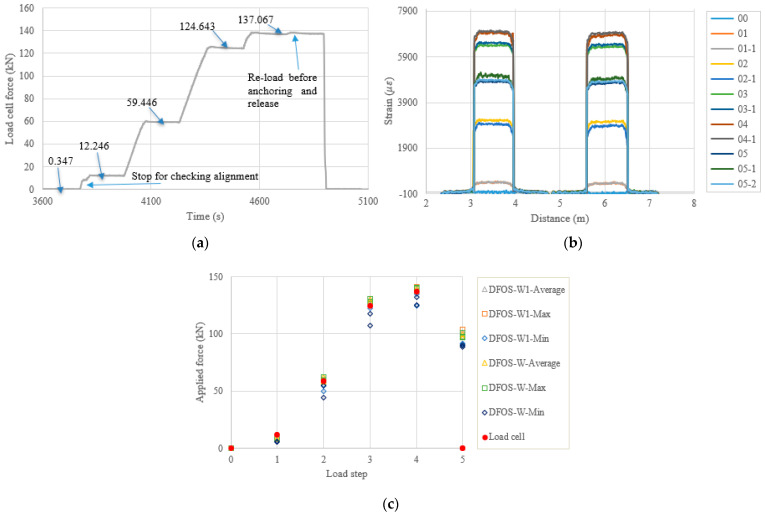
(**a**) Load cell readings for PC3-2; (**b**) strain distribution along the strand (W1 and W fibers) (In the legend, the first number represents the load step, and the second number represents measurement times at the load step); and (**c**) applied force changes at different load steps.

**Figure 13:**
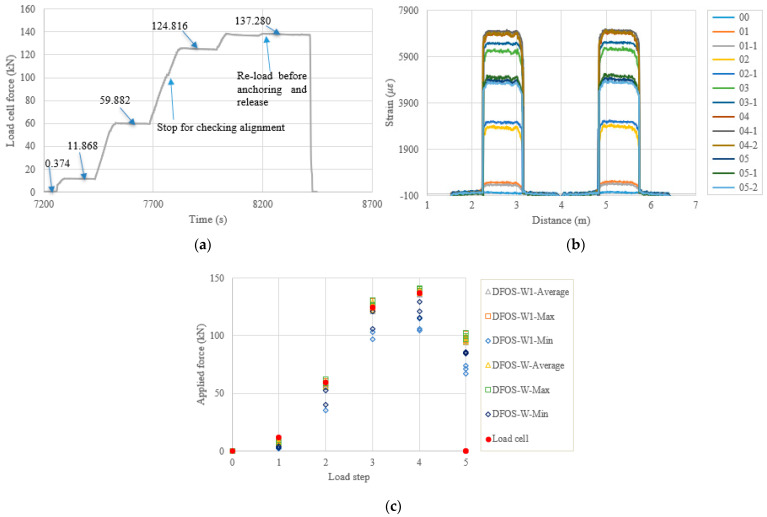
(**a**) Load cell readings for PC4-1; (**b**) strain distribution along the strand (W1 and W fibers) (In the legend, the first number represents the load step, and the second number represents measurement times at the load step); and (**c**) applied force changes at different load steps.

**Figure 14:**
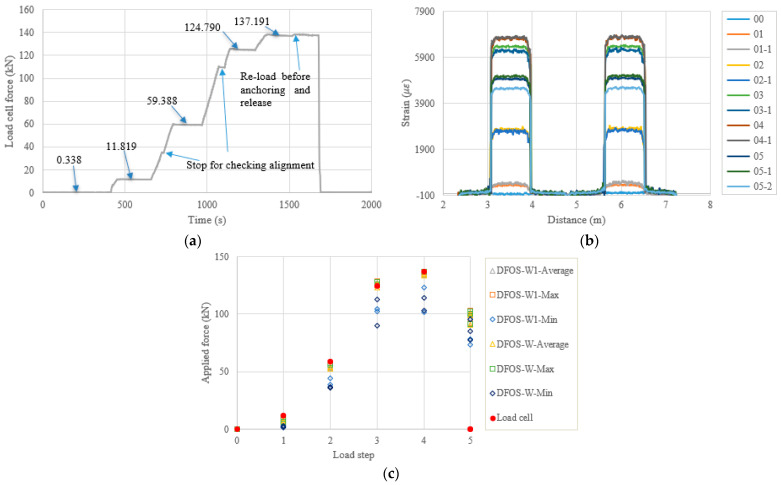
(**a**) Load cell readings for PC4-2; (**b**) strain distribution along the strand (W1 and W fibers) (In the legend, the first number represents the load step, and the second number represents measurement times at the load step); and (**c**) applied force changes at different load steps.

Figure 11, Figure 12, Figure 13 and Figure 14 and Table 8, Table 9, Table 10 and Table 11 show the testing results of the remaining four specimens. To compensate for the instant prestress loss after the force release, as well as to maintain a high prestress level in the strand, the applied force was increased by 10%, compared to the previous four specimens. Therefore, one more load level appears in Figure 11, Figure 12, Figure 13 and Figure 14a. However, the testing and calculated results presented in Figure 11, Figure 12, Figure 13 and Figure 14 are like the previous four specimens. Obviously, after the force release, the DFOSs measured higher strain levels than were measured for the previous four specimens, which had lower target loads. Moreover, the force errors between the load cell and the DFOSs were within approximately 5% for the average and maximum cases in these four specimens.

### 3.2. Linear Regression Analysis

The aim of the present study was to use DFOSs to monitor the prestressing force in cables. If a simple and direct relationship between the prestressing force and the measured quantities could be established, the DFOS-assisted cable force monitoring would have a more solid basis on which to promote its application in engineering structures. Based on the significant amount of data obtained from the DFOSs for the different specimens, the regression analysis could be a mathematical tool to investigate the relationship between the ground truth cable force and the DFOS strain (or calculated force). Here, the prestressing force in the cable from the load cell can be a dependent variable, while the independent variable can be the DFOS strain (or calculated force). Because only one independent variable exists, a linear regression model can be used to establish their relationship. The least square method is usually used to obtain a theoretical prediction equation describing this relationship. The principle of the least square method is to minimize the sum of the squared residuals.

**Figure 15:**
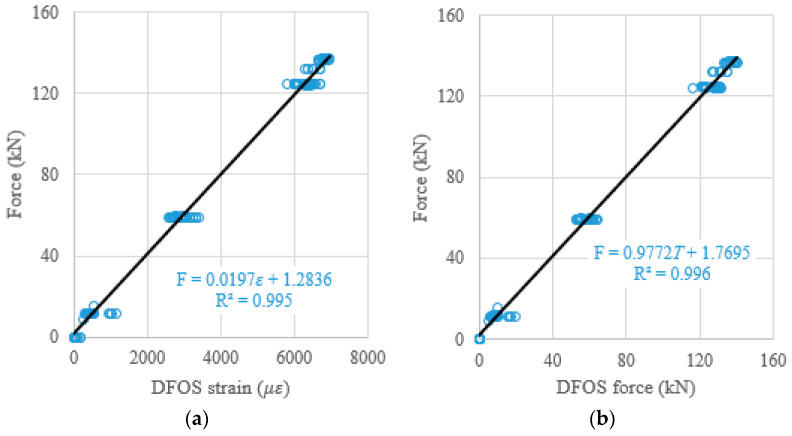
(**a**) Prestressing force versus DFOS average strain; and (**b**) prestressing force versus DFOS average strain-calculated force.

**Figure 16:**
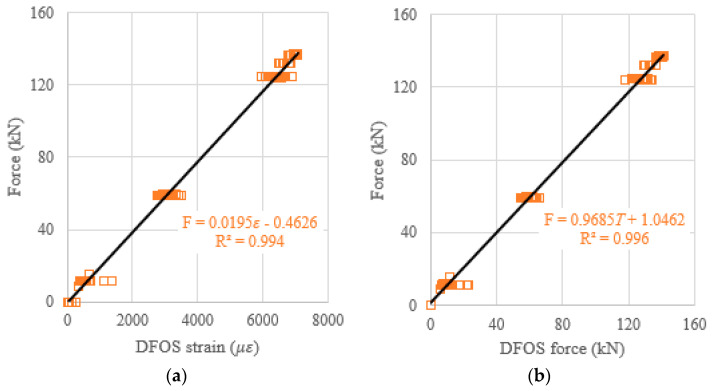
(**a**) Prestressing force versus DFOS-max. strain; and (**b**) prestressing force versus DFOS max. strain-calculated force.

**Figure 17:**
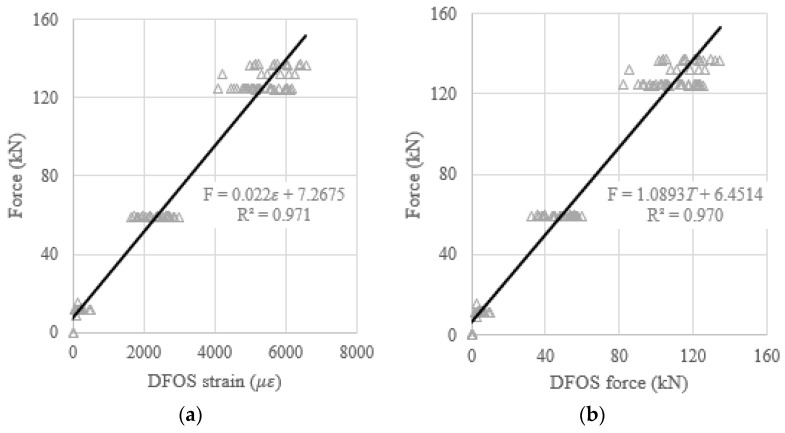
(**a**) Prestressing force versus DFOS-min. strain; and (**b**) prestressing force versus DFOS min. strain-calculated force.

When the DFOS average strain obtained from the strain distribution along the strand, or when the DFOS average strain-calculated force was used as an independent variable to predict the prestressing force (as a dependent variable), the theoretical prediction equations, using the least square method, were presented in Figure 15a,b. Moreover, to verify the effectiveness of the theoretical prediction equations, the determination coefficients (R^2^) were reported in Figure 15. It was found that the R^2^ are 0.995 for the average strain case, and 0.996 for the average strain-calculated force case. Therefore, the measured strain from the DFOSs show a strong correlation with the load cell force (i.e., ground truth) because the determination coefficients approach one. The same procedures are applied to the relations between the DFOS-max. strain and the cable force, or between the DFOS-min. strain and the cable force. For the max. strain and the max. strain-calculated force as independent variables, the R^2^ were 0.994 and 0.996, as shown in Figure 16, respectively. For the min. strain and the min. strain-based force as independent variables, the R^2^ were 0.971 and 0.970, as shown in Figure 17, respectively. In addition, it was observed that the line slopes in Figure 15b and Figure 16b were less than one, and less than the line slope in Figure 17b. All residual terms in the fitted equations were small and the residual terms in Figure 15 and Figure 16 were smaller than 2 kN.

### 3.3. Instant Prestress Loss

**Figure 18:**
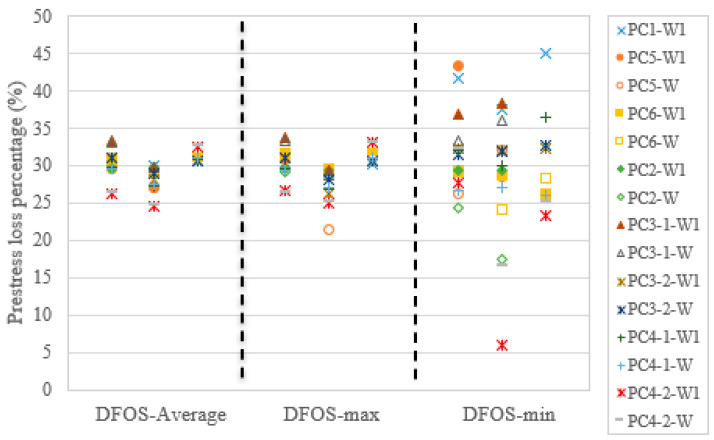
Prestress loss percentage for all specimens in the present test setup.

In this test setup, the load cell could not monitor the prestressing force after the force release. The DFOS provided useful strain data to quantify the instant prestress loss due to the anchor retraction, end steel plate and epoxy deformation, and the elastic deformation of the concrete specimen. As the DFOS strain or the DFOS strain-calculated force had a linear relationship with the cable force, as discussed in Section 3.2, the prestress loss percentage could be determined by the strain-calculated force change before and after the force release, which means that it is not necessary to know the actual cable force if only the prestress loss percentage is required. However, the actual cable force after the force release can be calculated by the linear equations, as shown in Figure 15, Figure 16 and Figure 17. To obtain the prestress loss percentage, the data in Table 4, Table 5, Table 6, Table 7, Table 8, Table 9, Table 10 and Table 11 were used. Here, the last-time DFOS measurement was taken before the force release was regarded as the reference value. Next, the instant prestress loss percentage was calculated by (the reference value − DFOS measured force after force release)/the reference value × 100%. Some specimens had two-time DFOS measurements and some specimens had three-time DFOS measurements. Moreover, for each specimen, there were two optic fibers (except for PC1) and three statistics (i.e., average strain, max. strain, and min. strain), which were used to calculate the DFOS-measured forces. Therefore, fifteen legends and three regions can be seen in Figure 18. Obviously, the scatter of prestress loss percentages can be observed for the different DFOS strain-calculated forces. The greatest scatter came from the DFOS-min. strain, followed by the DFOS-max. strain, and the DFOS average strain. For the average and max. cases, the prestress loss percentages were within 25–35% in this test setup. For example, if the target prestressing force is 65% of the ultimate strength of the steel strand, the remaining prestress level will be 45.5% after force release, with a prestress loss percentage of 30% (which is the case for PC1). For PC2, after the instant prestress loss, the prestress level was up to 51.6%.

## 4. Conclusions

The present study proposes a spiral deployment scheme of Brillouin scattering-based DFOSs to measure the prestressing forces of the cables in concrete bars, which were post-tensioned in a custom-built test setup in this study, to replace load cell because of the installation complexity and availability. The DFOSs were helically bonded to the steel strand, along the valley between the adjacent two outer wires. All the DFOSs survived during the tensioning process (up to 75% of the ultimate strength of the steel strand) and after the force release, and the strain distributions along the strand were obtained from the strain–frequency coefficient. After considering the bonded DFOS direction, along with the material properties of the strand, the cable forces could be calculated at different load levels. With the strain distribution along the strand, three statistics (i.e., DFOS-average strain, DFOS-maximum strain, and DFOS-minimum strain) were used to calculate the cables’ forces. Comparing the DFOS-measured cable forces (including average, max., and min.) with the load cell values (and oil pressure meter values for the PC1 specimen only), the relative errors between them at the small load level (less than 12 kN) were high (more than 50%), while the relative errors were smaller than approximately 10% after the second load level, and were even 5% for the average and max. cases in some specimens. For the three cases (i.e., average, max., and min.), the relationships between the DFOS-measured strains (or calculated forces) and the load cell forces were established. All of them showed good linearity with the minimum determination coefficient of 0.970, in the min. case. The residual terms were smaller than 2 kN in the average and max. cases. Moreover, based on the test DFOS results before and after the force release, the instant prestress loss percentages for the three cases were calculated. The most evident scatter was observed in the min. case, while approximately 30% instant prestress loss percentage could be determined from the average and max. cases for the present test setup. Furthermore, it was found that the epoxy type may have significantly affected the measured cable forces, which warrants further investigation. The epoxy construction procedure may have induced optical fiber position uncertainty and void defects near the bottom triangle region. One approach that could potentially solve the void defect could be for one thin layer of epoxy to be put at the valley first, as a cushion layer, and the optical fiber could then be placed as it was in the present study. The second layer of epoxy could then cover the top layer. However, robot-assisted automatic construction would still be desirable to reduce the optical fiber position uncertainty in the future. Moreover, the strain transfer from the steel wires to the optical fiber also deserves further investigation, since two steel wires are involved in this strain transfer and these steel wires have a spiral shape in space, which is different to traditional strain transfer cases.

## Figures and Tables

**Table 1:** Mix proportions of concrete.

Components	Content
Cement (kg/m^3^)	260
Fly ash (kg/m^3^)	110
River sand (kg/m^3^)	1020
Coarse aggregate (3/8–4/8 in) (kg/m^3^)	750
AEA (L/m^3^)	0.3
HRWR (L/m^3^)	2.6
VMA (L/m^3^)	3.3
BT50 fiber (kg/m^3^)	3.0

**Table 2:** Fiber properties.

Items	STRUX BT50
Material	Polypropylene
Shape	Straight
Color	White
Cross-section	Rectangle
Specific gravity	0.91
Length (mm)	50
Width (mm)	0.667
Aspect ratio	75
Thickness (mm)	0.25
Elastic modulus (GPa)	7
Tensile strength (MPa)	550
Absorption	None
Melting point	160 ℃
Ignition point	570 ℃
Alkali, acid, and salt resistance	High
Addition rate (kg/m^3^)	4.0–9.0

**Table 3:** Specimen design with different parameters.

Specimens	Bonded/Unbonded	Adhesives	Paper Tapes for Defects	Duct Materials
PC1	Bonded	Normal epoxy	No	Plastic
PC2	Unbonded	Normal epoxy	No	Metallic
PC3-1	Bonded	Normal epoxy	Yes	Plastic
PC3-2	Bonded	Normal epoxy	Yes	Plastic
PC4-1	Bonded	Normal epoxy	Yes	Metallic
PC4-2	Bonded	Normal epoxy	Yes	Metallic
PC5	Bonded	High temperature	No	Plastic
PC6	Bonded	Normal epoxy	No	Metallic

**Table 4:** Prestressing force monitored by load cell and DFOS for PC1 (unit: kN).

Load Step	Load Cell	DFOS Average	Error (%)	DFOS max.	Error (%)	DFOS min.	Error (%)
00	0.000	0.000	0.00	0.000	0.00	0.000	0.00
01	8.727	5.001	−42.69	5.837	−33.12	2.434	−72.12
02	11.658	9.469	−18.77	10.515	−9.81	3.714	−68.14
03	15.191	10.347	−31.89	11.887	−21.75	2.863	−81.15
04	58.878	57.741	−1.93	58.670	−0.35	50.976	−13.42
05	124.34	116.364	−6.41	118.274	−4.88	106.300	−14.51
06-1	n/a	80.858	n/a	82.366	n/a	61.910	n/a
06-2	n/a	81.419	n/a	83.813	n/a	66.515	n/a
06-3	n/a	80.724	n/a	82.532	n/a	58.54	n/a
00	0.000	0.000	0.00	0.000	0.00	0.000	0.00
06-3	n/a	82.727	n/a	85.102	n/a	71.228	n/a

**Table 5:** Prestressing force monitored by load cell and DFOS for PC5 (unit: kN).

Load Step	Load Cell	DFOS Average	Error (%)	DFOS max.	Error (%)	DFOS min.	Error (%)
00	0.000	0.000	0.00	0.000	0.00	0.000	0.00
01-1	11.321	19.442	71.74	22.767	101.10	8.980	−20.68
01-2	11.321	16.558	46.26	17.765	56.92	9.970	−11.93
02-1	58.975	64.646	9.62	65.826	11.62	59.927	1.61
02-2	58.975	63.286	7.31	63.763	8.12	57.593	−2.34
03-1	124.317	131.207	5.54	134.232	7.98	97.893	−21.26
03-2	124.317	123.198	−0.90	128.209	3.13	105.955	−14.77
04-1	n/a	86.255	n/a	88.745	n/a	60.102	n/a
04-2	n/a	89.928	n/a	95.567	n/a	75.732	n/a
00	0.000	0.000	0.00	0.000	0.00	0.000	0.00
01-1	11.321	17.596	55.43	22.303	97.01	3.986	−64.79
01-2	11.321	15.802	39.58	17.219	52.10	3.548	−68.66
02-1	58.975	63.789	8.16	64.648	9.62	53.265	−9.68
02-2	58.975	62.353	5.73	63.480	7.64	48.989	−16.93
03-1	124.317	131.386	5.69	133.635	7.50	96.236	−22.59
03-2	124.317	123.519	−0.64	128.161	3.09	99.415	−20.03
04-1	n/a	86.910	n/a	87.657	n/a	73.345	n/a
04-2	n/a	90.001	n/a	100.700	n/a	67.549	n/a
00	0.000	0.000	0.00	0.000	0.00	0.000	0.00

**Table 6:** Prestressing force monitored by load cell and DFOSs for PC6 (unit: kN).

Load Step	Load Cell	DFOS Average	Error (%)	DFOS max.	Error (%)	DFOS min.	Error (%)
00	0.000	0.000	0.00	0.000	0.00	0.000	0.00
01-1	11.470	9.778	−14.75	9.954	−13.22	4.793	−58.21
01-2	11.470	8.813	−23.17	9.050	−21.09	4.831	−57.88
02-1	59.228	52.684	−11.05	55.120	−6.94	38.269	−35.39
02-2	59.228	55.129	−6.92	55.558	−6.20	50.212	−15.22
03-1	124.565	129.824	4.22	129.990	4.36	123.070	−1.20
03-2	124.565	121.955	−2.10	122.225	−1.88	113.563	−8.83
03-3	124.565	121.583	−2.39	123.618	−0.76	106.149	−14.78
04-1	n/a	84.044	n/a	84.463	n/a	74.888	n/a
04-2	n/a	86.115	n/a	87.124	n/a	75.708	n/a
04-3	n/a	83.570	n/a	83.802	n/a	78.337	n/a
00	0.000	0.000	0.00	0.000	0.00	0.000	0.00
01-1	11.470	9.715	−15.30	9.610	−16.22	6.132	−46.53
01-2	11.470	8.760	−23.63	8.866	−22.70	5.474	−52.27
02-1	59.228	53.201	−10.18	54.809	−7.46	39.973	−32.51
02-2	59.228	54.715	−7.62	55.225	−6.76	48.161	−18.68
03-1	124.565	129.527	3.98	129.883	4.27	122.171	−1.92
03-2	124.565	121.545	−2.42	121.921	−2.12	114.089	−8.41
03-3	124.565	121.325	−2.60	123.061	−1.21	103.198	−17.15
04-1	n/a	83.614	n/a	84.368	n/a	74.428	n/a
04-2	n/a	85.748	n/a	86.576	n/a	78.267	n/a
04-3	n/a	83.220	n/a	83.619	n/a	73.952	n/a
00	0.000	0.000	0.00	0.000	0.00	0.000	0.00

**Table 7:** Prestressing force monitored by load cell and DFOSs for PC2 (unit: kN).

Load Step	Load Cell	DFOS Average	Error (%)	DFOS max.	Error (%)	DFOS min.	Error (%)
00	0.000	0.000	0.00	0.000	0.00	0.000	0.00
01	11.382	9.150	−19.61	10.798	−5.13	4.544	−60.08
02-1	59.231	53.503	−9.67	57.117	−3.57	43.485	−26.58
02-2	59.231	61.811	4.36	62.657	5.78	57.268	−3.31
03-1	124.798	130.694	4.72	132.112	5.86	124.189	−0.49
03-2	124.798	128.877	3.27	130.855	4.85	113.418	−9.12
04-1	n/a	90.736	n/a	92.226	n/a	80.010	n/a
04-2	n/a	90.401	n/a	92.339	n/a	80.158	n/a
00	0.000	0.000	0.00	0.000	0.00	0.000	0.00
01	11.382	9.439	−17.07	11.646	2.32	4.157	−63.47
02-1	59.231	54.073	−8.71	57.163	−3.49	45.479	−23.22
02-2	59.231	62.079	4.81	62.607	5.70	56.327	−4.90
03-1	124.798	130.545	4.61	132.029	5.79	92.752	−25.68
03-2	124.798	129.028	3.39	130.641	4.68	99.633	−20.16
04-1	n/a	90.975	n/a	92.668	n/a	75.315	n/a
04-2	n/a	90.958	n/a	93.059	n/a	82.293	n/a
00	0.000	0.000	0.00	0.000	0.00	0.000	0.00

**Table 8:** Prestressing force monitored by load cell and DFOSs for PC3-1 (unit: kN).

Load Step	Load Cell	DFOS Average	Error (%)	DFOS max.	Error (%)	DFOS min.	Error (%)
00	0.000	0.000	0.00	0.000	0.00	0.000	0.00
01-1	11.576	9.971	−13.87	11.075	−4.33	5.905	−48.99
01-2	11.576	9.007	−22.20	9.689	−16.30	5.580	−51.80
02-1	59.310	58.755	−0.94	60.802	2.52	48.038	−19.00
02-2	59.310	60.736	2.40	61.696	4.02	53.975	−9.00
03-1	124.596	127.729	2.52	130.092	4.41	93.497	−24.96
03-2	124.596	127.368	2.23	129.862	4.23	82.331	−33.92
04-1	131.969	131.521	−0.34	133.236	0.96	122.925	−6.85
04-2	131.969	134.823	2.16	136.900	3.74	85.188	−35.45
04-3	131.969	127.750	−3.20	130.275	−1.28	107.734	−18.36
05-1	n/a	85.194	n/a	86.390	n/a	68.108	n/a
05-2	n/a	89.592	n/a	92.081	n/a	66.347	n/a
00	0.000	0.000	0.00	0.000	0.00	0.000	0.00
01-1	11.576	9.694	−16.26	10.448	−9.75	4.537	−60.80
01-2	11.576	8.847	−23.58	9.330	−19.40	4.306	−62.80
02-1	59.310	59.566	0.43	66.369	11.90	44.219	−25.44
02-2	59.310	61.320	3.39	62.316	5.07	54.434	−8.22
03-1	124.596	127.729	2.52	128.928	3.48	117.508	−5.69
03-2	124.596	128.225	2.91	129.133	3.64	121.631	−2.38
04-1	131.969	131.081	−0.67	132.517	0.41	118.657	−10.09
04-2	131.969	135.008	2.30	136.341	3.31	126.597	−4.07
04-3	131.969	127.023	−3.75	129.567	−1.82	111.366	−15.61
05-1	n/a	85.028	n/a	86.403	n/a	74.242	n/a
05-2	n/a	89.335	n/a	92.386	n/a	71.173	n/a

**Table 9:** Prestressing force monitored by load cell and DFOSs for PC3-2 (unit: kN).

Load Step	Load Cell	DFOS Average	Error (%)	DFOS max.	Error (%)	DFOS min.	Error (%)
00	0.000	0.000	0.00	0.000	0.00	0.000	0.00
01-1	11.901	7.856	−33.98	7.778	−34.64	6.277	−47.26
01-2	11.901	8.167	−31.37	7.992	−32.85	6.109	−48.66
02-1	59.101	62.171	5.19	62.310	5.43	55.481	−6.13
02-2	59.101	58.867	−0.40	59.752	1.10	49.959	−15.47
03-1	124.296	128.155	3.10	128.271	3.20	122.666	−1.31
03-2	124.296	130.625	5.09	130.840	5.26	125.277	0.79
04-1	136.722	139.230	1.83	139.844	2.28	124.369	−9.04
04-2	136.722	140.397	2.69	140.861	3.03	134.637	−1.52
05-1	n/a	96.833	n/a	97.022	n/a	91.201	n/a
05-2	n/a	100.908	n/a	103.849	n/a	91.518	n/a
05-3	n/a	97.519	n/a	97.767	n/a	91.275	n/a
00	0.000	0.000	0.00	0.000	0.00	0.000	0.00
01-1	11.901	8.261	−30.58	8.604	−27.70	5.253	−55.86
01-2	11.901	8.534	−28.29	8.191	−31.17	5.653	−52.50
02-1	59.101	62.172	5.20	62.381	5.55	54.573	−7.66
02-2	59.101	58.101	−1.69	59.249	0.25	44.358	−24.94
03-1	124.296	127.818	2.83	128.181	3.13	117.758	−5.26
03-2	124.296	130.114	4.68	130.546	5.03	107.454	−13.55
04-1	136.722	137.864	0.84	139.582	2.09	125.185	−8.44
04-2	136.722	140.098	2.47	140.486	2.75	131.847	−3.57
05-1	n/a	96.690	n/a	96.889	n/a	90.255	n/a
05-2	n/a	99.559	n/a	101.081	n/a	89.953	n/a
05-3	n/a	97.331	n/a	97.415	n/a	88.762	n/a
00	0.000	0.000	0.00	0.000	0.00	0.000	0.00

**Table 10:** Prestressing force monitored by load cell and DFOSs for PC4-1 (unit: kN).

Load Step	Load Cell	DFOS Average	Error (%)	DFOS max.	Error (%)	DFOS min.	Error (%)
00	0.000	0.000	0.00	0.000	0.00	0.000	0.00
01-1	11.493	8.443	−26.54	8.822	−23.25	3.251	−71.72
01-2	11.493	6.530	−43.18	7.211	−37.26	1.927	−83.23
02-1	59.508	55.142	−7.34	57.827	−2.83	35.350	−40.60
02-2	59.508	60.493	1.65	61.206	2.85	55.365	−6.96
03-1	124.446	121.118	−2.67	124.099	−0.28	96.900	−22.13
03-2	124.446	128.895	3.58	130.266	4.68	102.899	−17.31
04-1	136.906	135.695	−0.88	138.568	1.21	104.218	−23.88
04-2	136.906	139.205	1.68	140.460	2.60	115.004	−16.00
04-3	136.906	136.206	−0.51	139.053	1.57	105.520	−22.92
05-1	n/a	95.657	n/a	97.962	n/a	71.612	n/a
05-2	n/a	98.979	n/a	101.689	n/a	73.822	n/a
05-3	n/a	94.209	n/a	96.033	n/a	67.085	n/a
00	0.000	0.000	0.00	0.000	0.00	0.000	0.00
01-1	11.493	8.601	−25.17	9.566	−16.77	4.360	−62.06
01-2	11.493	6.738	−41.38	7.461	−35.08	2.929	−74.52
02-1	59.508	55.741	−6.33	58.660	−1.43	39.918	−32.92
02-2	59.508	60.332	1.38	61.975	4.15	52.926	−11.06
03-1	124.446	122.215	−1.79	125.573	0.91	105.557	−15.18
03-2	124.446	129.420	4.00	130.457	4.83	120.906	−2.84
04-1	136.906	136.909	0.00	139.267	1.72	120.852	−11.73
04-2	136.906	139.613	1.98	141.103	3.07	129.390	−5.49
04-3	136.906	137.287	0.28	140.571	2.68	115.665	−15.52
05-1	n/a	96.637	n/a	99.383	n/a	84.826	n/a
05-2	n/a	99.644	n/a	102.654	n/a	84.301	n/a
05-3	n/a	94.752	n/a	96.981	n/a	85.673	n/a
00	0.000	0.000	0.00	0.000	0.00	0.000	0.00

**Table 11:** Prestressing force monitored by load cell and DFOSs for PC4-2 (unit: kN).

Load Step	Load Cell	DFOS Average	Error (%)	DFOS max.	Error (%)	DFOS min.	Error (%)
00	0.000	0.000	0.00	0.000	0.00	0.000	0.00
01-1	11.478	6.778	−40.95	7.475	−34.87	2.567	−77.64
01-2	11.478	7.767	−32.34	8.902	−22.44	2.328	−79.72
02-1	59.051	54.666	−7.43	57.674	−2.33	44.315	−24.95
02-2	59.051	53.455	−9.48	55.740	−5.61	38.914	−34.10
03-1	124.451	127.394	2.37	128.788	3.49	102.470	−17.66
03-2	124.451	123.359	−0.88	125.285	0.67	104.631	−15.93
04-1	136.853	134.335	−1.84	136.297	−0.41	122.983	−10.14
04-2	136.853	134.899	−1.43	137.109	0.19	101.760	−25.64
05-1	n/a	99.606	n/a	100.636	n/a	73.579	n/a
05-2	n/a	101.721	n/a	102.959	n/a	95.737	n/a
05-3	n/a	90.972	n/a	91.713	n/a	78.140	n/a
00	0.000	0.000	0.00	0.000	0.00	0.000	0.00
01-1	11.478	5.988	−47.83	6.562	−42.83	2.729	−76.22
01-2	11.478	7.408	−35.47	9.292	−19.05	1.549	−86.50
02-1	59.051	54.246	−8.14	57.732	−2.23	36.300	−38.53
02-2	59.051	52.863	−10.48	55.910	−5.32	36.627	−37.97
03-1	124.451	126.666	1.78	128.316	3.11	90.153	−27.56
03-2	124.451	123.117	−1.07	125.757	1.05	112.596	−9.53
04-1	136.853	133.648	−2.34	136.684	−0.12	102.903	−24.81
04-2	136.853	134.723	−1.56	136.714	−0.10	114.171	−16.57
05-1	n/a	99.201	n/a	100.586	n/a	77.373	n/a
05-2	n/a	101.354	n/a	102.400	n/a	95.209	n/a
05-3	n/a	90.596	n/a	91.363	n/a	85.452	n/a
00	0.000	0.000	0.00	0.000	0.00	0.000	0.00

## Data Availability

Data is contained within the article.
